# Supplementary material for: The Association of Parental Interest in Entrepreneurship with the Entrepreneurial Interest of Spanish Youth
Source: Int J Environ Res Public Health. 2020 Jul 1;17(13):4744. doi: 10.3390/ijerph17134744 (PMC7369814; doi:10.3390/ijerph17134744)
Supplement: Supplementary file 1 [file ijerph-17-04744-s001.zip › S1_Student Questioonnaire_English.pdf]

Unión Europea

Fondo Europeo de Desarrollo Regional

FINANCED UNDER THE NATIONAL PLAN I+D+i (2012-2015)

Codes: EDU2012-39080-C07-00

EDU2012-39080-C07-01 | EDU2012-39080-C07-05
EDU2012-39080-C07-03 | EDU2012-39080-C07-06
EDU2012-39080-C07-04 | EDU2012-39080-C07-07

QUESTIONNAIRE FOR STUDENTS

From educational times to social times: the daily construction of the situation of young people in a networked society. Social and teaching-related problems and alternatives.

This anonymous and confidential questionnaire is designed to find out how you organize and share academic and leisure time in your daily life. Your collaboration is essential for the development of this project, for which reason we are asking you to give us a few minutes of your time and respond sincerely to all the questions. Many Thanks.

STUDENTS

1. Sex: oWoman oMan

2. Year of birth:

3. Indicate the people who live at your home (your normal place of residence). *Mark as many as necessary*
oMother oFather oTutor(s) oBrother/Sister(s) oPartner of your mother/father o Grandmother/father(s) oOther family members oFellow students o*Others Who?*

4. Counting yourself, there are .... people living at your home

5. Indicate the answer that reflects your family situation
oYour parents live together oYour parents live apart oYou only have a mother oYou only have a father oYou have neither a father nor a mother o*Other What?*

6. Indicate your relation with your family: oNatural oAdopted oFoster **Son/Daughter**

7. What country is your family from?

	COUNTRY OF BIRTH	NATIONALITY		
		Spanish	Foreigner	Dual
Your father		o	o	o
Your mother		o	o	o
Yourself		o	o	o

8. Indicate postcode................................ and name of the place (city/town/village) where you live

9. How much money do have to spend each week? €................................

10. What is your level of studies and what is the employment situation of your parents?

	Studies				Employment situation					
	None	Primary (EGB)	Secondary (Bachiller, FP)	Higher Education/ University	Employee	Self-employed/ Entrepreneur	Home care	No Job	Retired	Pensioner
**Father**	o	o	o	o	o	o	o	o	o	o
**Mother**	o	o	o	o	o	o	o	o	o	o

LIFE AT THE EDUCATIONAL CENTER

11. You normally travel to your educational center: *Mark only one option*

○Walking – ○By bicycle – ○By motorbike – ○By school bus – ○On public transport – ○By car (I'm driven) - ○By car (I drive)

○Other, which? ....

12. How long does it usually take you to travel from your home to your educational center? .... minutes

13. Indicate your agreement with the following expressions *(1= not at all, 2=a little, 3=somewhat, 4=quite a lot* and *5=very much so)*

CLASS DAYS ...		1-5
It is difficult for me to maintain my attention in class.		
I feel weighed down everything that I have to do.		
I feel tired when the classes are over.		
I have enough time	to be with my friends	
	to be with my family	
	to do sports	
	to go to extra-curricular activities	
	to do the things I like	
Time passes very quickly at the educational center		
Time passes very quickly outside the educational center		

14. How often can you do the following in your classes? *(1= not at all, 2=a little, 3=somewhat, 4=quite often* and *5=very often)*

	1-5
Work in groups and work collaboratively	
Use other spaces and resources of the center (library, computer and ICT room, etc.)	
Work at my own pace	
Alternate between activities of different intensity (activity/rest)	
Listen to the explanations of the teacher	
Reflect upon and discuss the subject matter	
Reflect upon and discuss topics in the news	
Organize things myself	
Speak about the problems that affect us	
Strengthen creativity	
Strengthen self-knowledge: preferences, likes, limits, etc.	
Comment on future training and/or professional careers	

15. since you have been in Non-obligatory Secondary Education *(**since you left ESO Escuela Secundaria Obligatoria****)*, what mark has been the most frequent one in your exams?** *(a figure between 0 and 10)* ....

16. What timetable do you prefer? *Mark only one option*

○Morning classes only
○Afternoon classes only
○Space classes between mornings and one afternoon
○Space classes between mornings and some afternoons
○Space classes between all the mornings and all the afternoons
○Other. Which? ....
....
○Makes no difference to you

17. How many minutes do you spend studying and doing homework every day? .... minutes

This time... ○Is too little ○Is enough ○Is excessive

18. Indicate your degree of satisfaction with the duration and the distribution of the classroom hours, breaks between classes, and holidays *(1= not at all, 2=a little, 3=somewhat, 4=quite a lot,* and *5=very satisfied). Point out also which aspects, if you could decide, you would modify*

		Satisfaction (1-5)	REDUCE	MAINTAIN	INCREASE
**Duration**	Class sessions		○	○	○
	Break times		○	○	○
	Vacations		○	○	○

		CONCENTRATE MORE	MAINTAIN	DISTRIBUTE
**Distribution**	Class sessions	○	○	○
	Break times	○	○	○
	Vacations	○	○	○

19. During the breaks... *Choose only one option (the most frequent) in each case*

- WHERE ARE YOU? ○Playground ○Library ○Canteen at center ○Street
  ○Other, Which? ....
- WHAT ARE YOU DOING? ○Going around with friends ○Doing sport ○Walking around ○Smoking
  ○Other, Which? ....

FAMILY LIFE

**20. Indicate your degree of agreement with the following expressions** *(1= not at all, 2=a little, 3=somewhat, 4=quite a lot and 5=very much so)*	**1-5**
My family life is ideal most of the time	
The conditions of my family life are excellent	
I am satisfied with my family life	
Up until now I have achieved the important things that I want to achieve in my family life	
If I were to decide about my family life, I would not change a thing	
One day I would like to have my own family life	

FREE TIME

21. Indicate the three most important leisure activities from among those you practice

a)

From among the following characteristics, mark with an "x" those that fit best with each of your preferred leisure activities

		Leisure activity				
		a	b	c	d	e
**23. Organization**	Independently, by myself					
	In an organized way, in an association, club, etc.					
	Both					
	Only in holiday periods.					
**24. Frequency**	Only 1-2 days a month.					
	Every week – Monday to Friday – 1-2 days					
	Every week – Monday to Friday – 3-4 days					
	Every week – Monday to Friday – 5 days					
	Every week – Weekends – 1 day					
	Every week – Weekends – 2 days					
**25. Company**<br>With whom do you do this activity?<br>*(mark as many options as necessary)*	Alone					
	With family					
	With people my age					
	With other people					
**26. Place**<br>Where you usually practice this activity?<br>*(point out as many as necessary)*	In private premises, lent or rented, that I share with people of my age					
	In associations, clubs, cultural groups (peñas), with people of different ages.					
	In municipal premises, such as sports halls, cultural centers play areas, etc.					
	In open public spaces such as the street, a park, a square, etc.					
	In leisure areas such as shopping malls, areas with bars, cinemas, discos, etc.					
	In the country (mountains, countryside, beach, sea, etc.)					
	In the installations of my own educational center					
	In houses, urbanizations and other private residences					
**27. If you have a digital device (smartphone, iPod, tablet, GPS, etc.) when on one of these activities, mark the corresponding box**						
**28. If you share and/or disseminate each one of these activities through the social media and/or virtual forums (wikis, blogs, Facebook, Twitter, etc.), tick the relevant box**						
**29. If you participate in any type of competition related with one of these activities (tournaments, competitions, contests, etc.), tick the relevant box**						

30. FROM THIS POINT, answer each question with a number from 1 to 5 *(1= not at all, 2=a little, 3=somewhat, 4=quite a lot, and 5=very much so)*

**31. Indicate the extent of your agreement with the following statements. When I am doing this activity....**	Leisure activity a	b	c	d	e
I am more in form, I control my movements better, I maintain and improve my physical condition, etc.					
I feel more satisfied, I enjoy doing it, I have fun, etc.					
I am more creative, I acquire knowledge, learn things, broaden my world, etc.					
I develop new manual skills and learn or get better at technical skills					
I do different things with more people, I feel part of a group, it helps me relate better to other people, etc.					

**32. Indicate the extent of your agreement with the following affirmations**	Leisure activity a	b	c	d	e
I hold responsibility in the organization for the activity					
I would enjoy the activity more if I took a leading role in its organization					
Participation in the organization of the space in which I practice the activity is related to what I get out of it.					
I practice this activity in spaces that I organize myself					
I am satisfied with this activity					
This activity is very important in my life					

33. Would you give up some leisure activity that you like? oYes oNo

If affirmative indicate which one: ....

34. If affirmative, what is your reason for doing it? *Mark only one option*

oLack of time
oLack of money
oNo partner
oNo where to do it in my area
oUndecidedness, laziness
oBecause I have a lot of homework
oMy family doesn't let me
oIncompatible hours
oTransport difficulties

oOther causes. Which? ....

HEALTH AND QUALITY OF LIFE

34. Indicate how many days a week (Monday to Sunday) you go for a walk and for how long.

Stroll or walk.. days a week and I usually spend... minutes each time.

35. How many hours do you usually sleep?

Days with class.. Weekends ....

**36. Indicate your degree of agreement with the following expressions** *(1=not at all, 2=a little, 3=somewhat, 4=quite a lot*, and *5=very much so)*	**1-5**
At present, my state of health is good	
I am satisfied with my corporal image	
I am in good physical shape	
I am a physically active person	
In general, I am enjoying my life	

STUDIES AND THE JOB MARKET IN THE FUTURE

37. Indicate how much you agree with the following *(1=not at all, 2=a little, 3=somewhat, 4=quite a lot*, and *5=a great deal)*

**Studying helps me to ...**	**1-5**	**Working helps me to...**	**1-5**
Be successful in life		Become independent	
Find work		Collaborate with my family	
Relate to colleagues at work		Feel useful	
Earn money		Have money	
I prefer to look for work than to continue studying		It is worth looking for work	

ENTREPRENEURSHIP

Entrepreneurship is defined as "creating your own firm or self-employment, in other words, detecting a business opportunity, organizing the necessary resources and assuming risks to contribute to your own professional and socio-economic development"

38. Bearing in mind the above definition, mark with an "x" the three aspects that you consider most important for entrepreneurship

Detect a business opportunity		Self-employment		Manage resources (human, economic...)	
Improve your professional development		Assume risk		Create new jobs	

Other relevant aspects. Indicate which: ....

39. Of the subjects that you have studied or are studying, which do you think favor entrepreneurship most of all? Indicate 3

40. Mark from 1 to 5 your degree of personal interest in entrepreneurship *(1= not at all, 2=a little, 3=somewhat, 4=quite a lot and 5=very much*

41. Do you know of some entrepreneurial network? oNo oYes, Which? ....

....

42. Mark with an "x" the three reasons that you consider most important to create your firm

Family tradition		Create work for others		Earn money	
Economic independence		Get a job		Put my ideas into practice	
Social acknowledgement		Group management		Contribute to economic and social growth	

Others. Indicate which: ....

43. Mark with an "x" the three most important difficulties that you perceive in society to create your own firm

Assume risks		Work too many hours		Lack of money to start up the activity	
Weak institutional support		Fear of failure		Little or no training in entrepreneurship	
Assume risks		Bureaucracy *(paperwork)*		Lack of ideas to start up the firm	
Weak institutional support		Others. Indicate which:			

44. Grade the presence that the following aspects have had in your training *(1=not at all, 2=a little, 3=somewhat, 4=quite a lot, and 5=very much so)*

**METHODOLOGIES**	**1-5**	**CAPABILITIES**	**1-5**	**KNOWLEDGE**	**1-5**
Theoretical classes		Leadership		Firm plan	
Practical exercises		Commitment and motivation		*Marketing*	
Case studies		Creativity/Innovation		Languages	
Project design		Conflict/crisis management (working under pressure)		Computing	
Working in a team		Communicative capability		Organizational planning	
Problem solving		Capability to negotiate and to take decisions		Obtaining resources	
Presentation and defense of works		Time management for personal and team work		Legal aspects of creating firms	
		Capacity to search for resources		Administrative management	

Observations. If you wish to take any notes, please use this space:

THANK YOU VERY MUCH FOR YOUR HELP

Red de Grupos de Investigación OcioGune

Universidades

Santiago de Compostela, Barcelona, Burgos, Deusto, La Rioja, Nacional de Educación a Distancia, Valéncia.

Contact: angela.devalenzuela@usc.es
