# Supplementary material for: The Association of Parental Interest in Entrepreneurship with the Entrepreneurial Interest of Spanish Youth
Source: Int J Environ Res Public Health. 2020 Jul 1;17(13):4744. doi: 10.3390/ijerph17134744 (PMC7369814; doi:10.3390/ijerph17134744)
Supplement: Supplementary file 1 [file ijerph-17-04744-s001.zip › S2_Student Questionnaire_Spanish.pdf]

OcioGune | resortes proyecto I+D+i
Red de Investigación en Ocio

MINISTERIO DE ECONOMÍA Y COMPETITIVIDAD

Unión Europea
Fondo Europeo de Desarrollo Regional

FINANCIADO POR EL PLAN NACIONAL I+D+i (2012-2015)

Códigos: **EDU2012-39080-C07-00**

EDU2012-39080-C07-01 | EDU2012-39080-C07-05
EDU2012-39080-C07-03 | EDU2012-39080-C07-06
EDU2012-39080-C07-04 | EDU2012-39080-C07-07

CUESTIONARIO PARA EL ALUMNADO

De los tiempos educativos a los tiempos sociales: la construcción cotidiana de la condición juvenil en una sociedad de redes. Problemáticas y alternativas pedagógico-sociales.

Este cuestionario, anónimo y confidencial, pretende conocer cómo organizas y compaginas los tiempos académicos y de ocio en tu día a día. Tu colaboración es imprescindible para desarrollar este trabajo, por lo que solicitamos que nos dediques unos minutos y respondas con sinceridad a todas las preguntas.
Muchas gracias.

ALUMNADO

1. Sexo: ☐ Mujer ☐ Hombre

2. Año de nacimiento:

3. Señala las personas que viven en tu residencia habitual. *Marca todas las que necesites*
☐ Madre ☐ Padre ☐ Tutor(es) ☐ Hermano(s) ☐ Pareja de tu madre/padre ☐ Abuela(o/s) ☐ Otros familiares ☐ Compañeros de estudios
☐ Otros ¿quiénes? ....

4. Contando contigo, sois en total .... **personas en tu residencia habitual**

5. Señala la respuesta que se corresponda con tu situación familiar
☐ Tus padres viven juntos ☐ Tus padres viven separados ☐ Sólo tienes madre ☐ Sólo tienes padre ☐ No tengo padre ni madre
☐ Otra ¿cuál? ....

6. Selecciona tu condición dentro de tu familia actual. Hijo/a: ☐ Biológico/a ☐ Adoptivo/a ☐ De acogida

7. ¿Cuál es el país de procedencia de tu familia?

	PAIS DE NACIMIENTO	NACIONALIDAD		
		Española	Extranjera	Doble
Tu padre		☐	☐	☐
Tu madre		☐	☐	☐
Tú		☐	☐	☐

8. Indica el código postal .... **y el nombre de la población en la que vives** ....

9. ¿De cuánto dinero dispones semanalmente para tus gastos? .... €

10. ¿Cuál es el nivel de estudios y la situación profesional de tus padres?

	Estudios				Situación Profesional					
	Ninguno	Primarios (EGB)	Secundarios (Bachiller, FP)	Superiores Universitarios	Empleado/a cuenta ajena	Trabajador/a cuenta propia (autónomo, empresario)	Atención del hogar	Paro	Jubilado/a	Pensionista
**Padre**	☐	☐	☐	☐	☐	☐	☐	☐	☐	☐
**Madre**	☐	☐	☐	☐	☐	☐	☐	☐	☐	☐

VIDA EN EL CENTRO ESCOLAR

11. Normalmente te desplazas a tu centro educativo: *Marca una sola opción*
☐ Andando ☐ En bicicleta ☐ En moto ☐ En autobús escolar ☐ En transporte público ☐ En coche (me llevan) ☐ En coche (conduzco)
☐ Otro, ¿cuál? ....

12. ¿Cuántos minutos tardas habitualmente en llegar desde casa a tu centro educativo? .... minutos

13. Indica tu grado de acuerdo con las siguientes expresiones *(1= nada, 2=poco, 3=algo, 4=bastante y 5=mucho)*

LOS DÍAS DE CLASE…		1-5
Me cuesta mantener la atención en las clases		
Me siento agobiada/o por todas las cosas que tengo que hacer		
Me siento fatigada/o al acabar las clases		
Tengo tiempo suficiente para	estar con mis amigas o amigos	
	estar con mi familia	
	practicar deporte	
	ir a actividades extraescolares	
	hacer las cosas que me gustan	
El tiempo que paso en el centro se me hace corto		
El tiempo que paso fuera del centro se me hace corto		

14. ¿Con qué frecuencia puedes realizar lo siguiente en tus clases? *(1= nada, 2=poco, 3=algo, 4=bastante y 5=mucho)*

	1-5
Trabajar en grupos y colaborativamente	
Utilizar otros espacios y recursos del centro (biblioteca, aula TIC, etc.)	
Trabajar a mi ritmo	
Alternar actividades de diversa intensidad (actividad/descanso)	
Escuchar las explicaciones del profesor/a	
Reflexionar y debatir sobre el temario	
Reflexionar y debatir sobre temas de actualidad	
Organizarme por mi cuenta	
Hablar sobre los problemas que nos afectan	
Potenciar la creatividad	
Potenciar el autoconocimiento: preferencias, gustos, límites, etc.	
Comentar sobre futuras orientaciones formativas y/o profesionales	

15. Desde que estás en Educación Secundaria Postobligatoria *(desde que dejaste la ESO),*
¿cuál es la nota que se repite más en tus evaluaciones? *(pon una cifra entre 0 y 10)* ....

16. ¿Qué modalidad de horario prefieres? *Marca una sola opción*

☐ Tener clase sólo por las mañanas
☐ Tener clase sólo por las tardes
☐ Repartir las clases entre todas las mañanas y una tarde
☐ Repartir las clases entre todas las mañanas y varias tardes
☐ Repartir las clases entre todas las mañanas y todas las tardes
☐ Otra. ¿Cuál? ....
....
☐ Te resulta indiferente

17. ¿Cuántos minutos dedicas diariamente a estudiar y realizar las tareas de clase? .... minutos
Este tiempo… ☐ Es escaso ☐ Está bien ☐ Es excesivo

18. Indica tu grado de satisfacción con la duración y distribución de las sesiones de clase, los recreos y vacaciones
(1= nada, 2=poco, 3=algo, 4=bastante y 5=mucho). Señala también qué aspectos modificarías o mantendrías si pudieras decidir

		Satisfacción (1-5)	REDUCIR	MANTENER	AUMENTAR
Duración	Sesiones clase		☐	☐	☐
	Recreos		☐	☐	☐
	Vacaciones		☐	☐	☐

			CONCENTRAR MÁS	MANTENER	DISTRIBUIR
Distribución	Sesiones clase		☐	☐	☐
	Recreos		☐	☐	☐
	Vacaciones		☐	☐	☐

19. Durante los recreos… *Escoge una única opción (la más frecuente) en cada caso*

- ¿DÓNDE ESTÁS? ☐ Patio ☐ Biblioteca ☐ Cafetería del centro ☐ Calle
☐ Otro, ¿cuál? ....

- ¿QUÉ HACES? ☐ Estar con amigas o amigos ☐ Hacer deporte ☐ Pasear ☐ Fumar
☐ Otra, ¿cuál? ....

VIDA FAMILIAR

**20. Indica tu grado de acuerdo con las siguientes expresiones** *(1= nada, 2=poco, 3=algo, 4=bastante y 5=mucho)*	1-5
La mayoría de las veces mi vida familiar es ideal	
Las condiciones de mi vida familiar son excelentes	
Estoy satisfecho con mi vida familiar	
Hasta ahora he conseguido las cosas importantes que quiero en mi vida familiar	
Si pudiera decidir sobre mi vida familiar, no cambiaría nada	
Algún día me gustaría tener mi propia familia	

TIEMPO LIBRE

21. De todas las actividades de ocio que practicas, indica por orden las 3 más importantes

a)

b)

c)

Si no has incluido ninguna actividad de ocio físico-deportivo, indica la que más practicas

d)

Si no has indicado ninguna actividad de ocio cultural, indica la que más realizas

e)

				Actividad de ocio a	b	c	d	e
**22. Duración** Cada vez que realizas esta actividad, ¿cuántos **minutos** le dedicas?								
**De las características señaladas a continuación, marca con una "x" las que mejor se ajusten a cada una de tus actividades de ocio preferidas**								
**23. Organización**	De forma libre, por mi cuenta							
	De forma organizada, en una asociación, club, etc.							
	Ambas							
**24. Frecuencia**	Sólo en períodos vacacionales							
	Sólo 1-2 días al mes							
	Todas las semanas	Lunes a viernes	1-2 días					
			3-4 días					
			5 días					
		Fines de semana	1 día					
			Los 2 días					
**25. Compañía** ¿Con quién practicas esta actividad? *(señala cuantas opciones necesites)*	Solo							
	Con mi familia							
	Con gente de mi edad							
	Con otras personas							
**26. Lugar de realización** ¿Dónde sueles practicar esta actividad? *(señala cuantas opciones necesites)*	En locales privados, cedidos o alquilados, que comparto con gente de mi edad							
	En asociaciones, clubes, sociedades o peñas, donde hay gente de distintas edades							
	En espacios municipales como polideportivos, centros culturales, ludotecas, etc.							
	En espacios públicos abiertos como la calle, un parque, una plaza, etc.							
	En espacios de ocio como centros comerciales, zonas de bares, cines, discotecas, etc.							
	En la naturaleza (montaña, campo, playa, mar, etc.)							
	En las instalaciones de mi propio centro escolar							
	En casas, urbanizaciones u otras residencias privadas							
**27. Si usas algún dispositivo digital (smartphone, iPod, tablet, receptor GPS, etc.) durante la realización de cada una de estas actividades, marca la casilla correspondiente**								
**28. Si compartes y/o difundes cada una de estas actividades a través de redes sociales y/o foros virtuales (wikis, blogs, Facebook, Twitter, etc.), marca la casilla correspondiente**								
**29. Si realizas algún tipo de competición relacionada con cada una de estas actividades (torneos, concursos, certámenes, etc.), marca la casilla correspondiente**								

A PARTIR DE AQUÍ responde a cada pregunta con un número del 1 al 5 *(1=nada, 2=poco, 3=algo, 4=bastante y 5=mucho)*

**30. Indica tu grado de acuerdo con las siguientes afirmaciones. Cuando realizo esta actividad...**	Actividad de ocio a	b	c	d	e
Estoy más en forma, controlo mejor mis movimientos, mantengo o mejoro mi condición física, etc.					
Me siento más satisfecho/a, disfruto haciéndolo, me divierte, etc.					
Soy más creativo/a, adquiero conocimientos, aprendo cosas, amplío mi mundo, etc.					
Desarrollo nuevas destrezas manuales y adquiero o perfecciono habilidades técnicas					
Hago cosas diferentes con más gente, me siento parte de un grupo, me ayuda a relacionarme mejor con los demás, etc.					

**31. Indica tu grado de acuerdo con las siguientes afirmaciones**	Actividad de ocio a	b	c	d	e
Tengo responsabilidad en la organización de la actividad					
Disfrutaría más de la actividad si asumiera más protagonismo en su organización					
Participar en la organización del espacio en el que practico la actividad tiene relación con los beneficios que saco de ella					
Practico esta actividad en espacios que yo mismo organizo					
Estoy satisfecho con esta actividad					
Esta actividad es muy importante en mi vida					

32. ¿Renuncias a realizar alguna actividad de ocio que te gusta? ☐ Sí ☐ No

En caso afirmativo indica cual: ....

33. En caso afirmativo, ¿por qué motivo lo haces? *Señala una sola opción de respuesta.*

☐ Falta de tiempo ☐ No hay donde hacerlo en mi localidad ☐ No me deja mi familia

☐ Falta de dinero ☐ Falta de decisión, pereza ☐ Horarios incompatibles

☐ No tengo con quién ☐ Porque tengo muchas tareas escolares ☐ Dificultades de transporte

☐ Otras causas. ¿Cuáles? ....

....

SALUD Y CALIDAD DE VIDA

34. Indica cuántos días a la semana (de lunes a domingo) caminas o paseas y cuánto tiempo le dedicas

Paseo o camino .... días a la semana y suelo dedicarle .... minutos cada vez

35. ¿Cuántas horas duermes habitualmente?

Los días de clase .... Los fines de semana ....

**36. Indica tu grado de acuerdo con las siguientes expresiones** *(1= nada, 2=poco, 3=algo, 4=bastante y 5=mucho)*	**1-5**
Actualmente, mi estado de salud es bueno	
Estoy satisfecho/a con mi imagen corporal	
Estoy en buena forma física	
Soy una persona físicamente activa	
En general, estoy a gusto con mi vida	

ESTUDIOS Y MERCADO LABORAL EN EL FUTURO

37. Indica tu grado de acuerdo con las siguientes expresiones *(1= nada, 2=poco, 3=algo, 4=bastante y 5=mucho)*

**Estudiar me ayuda a…**	**1-5**	**Trabajar me ayuda a…**	**1-5**
Tener éxito en la vida		Independizarme	
Encontrar trabajo		Colaborar con la familia	
Relacionarme con los compañeros/as		Sentirme útil	
Ganar dinero		Disponer de dinero	
Prefiero buscar trabajo a seguir estudiando		Merece la pena buscar trabajo	

EMPRENDIMIENTO

El emprendimiento se define como "crear tu propia empresa o autoemplearte, es decir, detectar una oportunidad de negocio, organizar los recursos necesarios y asumir riesgos para contribuir a tu desarrollo profesional y socioeconómico"

38. De la definición anterior, señala con una "x" los tres aspectos que consideras más importantes para el emprendimiento

Detectar una oportunidad de negocio		Autoemplearte		Gestionar recursos (humanos, económicos...)	
Mejorar tu desarrollo profesional		Asumir algún riesgo		Generar nuevos puestos de trabajo	

Otros aspectos relevantes. Indica cuáles: ....

39. De las asignaturas que cursas o has cursado ¿cuáles crees que favorecen más el emprendimiento? Indica 3

40. Valora de 1 a 5 tu grado de interés personal por el emprendimiento *(1= nada, 2=poco, 3=algo, 4=bastante y 5=mucho)*

41. ¿Conoces alguna red de emprendimiento? ☐ No ☐ Sí, ¿cuál o cuáles? ....

....

42. Señala con una "x" los tres motivos que consideras más importantes para crear tu empresa

Tradición familiar		Generar trabajo para otros		Ganar dinero	
Independencia económica		Conseguir trabajo		Poner en práctica mis ideas	
Reconocimiento social		Dirigir un grupo humano		Contribuir al crecimiento económico y social	

Otros. Indica cuáles: ....

43. Señala con una "x" las tres dificultades más importantes que percibes en la sociedad para crear tu propia empresa

Asumir riesgos		Trabajar demasiadas horas		Falta de dinero para iniciar la actividad	
Escaso apoyo institucional		Temor a fracasar		Deficiente formación recibida para emprender	
Imagen del empresario		Excesiva burocracia *(papeleo)*		Carencia de ideas para iniciar la empresa	
Poco apoyo familiar		Otros. Indica cuáles:			

44. Valora la presencia que han tenido los siguientes aspectos en tu formación *(1= nada, 2=poco, 3=algo, 4=bastante y 5=mucho)*

**METODOLOGÍAS**	**1-5**	**CAPACIDADES**	**1-5**	**CONOCIMIENTOS**	**1-5**
Clases teóricas		Liderazgo		Plan de empresa	
Ejercicios prácticos		Compromiso y motivación		*Marketing*	
Estudios de caso		Creatividad/Innovación		Idiomas	
Diseño de Proyectos		Gestión de conflictos/crisis (tolerancia a la presión)		Informática	
Trabajo en equipo		Capacidad comunicativa		Planificación de organizaciones	
Resolución de problemas		Capacidad de negociación y toma de decisiones		Obtención de recursos	
Exposición y defensa de trabajos		Gestión del tiempo para el trabajo propio y del equipo		Aspectos legales de creación de empresas	
		Capacidad de búsqueda de recursos		Gestión administrativa	

Observaciones. Si quieres realizar alguna anotación utiliza el espacio siguiente:

MUCHAS GRACIAS POR TU COLABORACIÓN

Red de Grupos de Investigación OcioGune

Universidades

Santiago de Compostela, Barcelona, Burgos, Deusto, La Rioja, Nacional de Educación a Distancia, Valéncia.

Contacto: angela.devalenzuela@usc.es
