# Supplementary material for: The Association of Parental Interest in Entrepreneurship with the Entrepreneurial Interest of Spanish Youth
Source: Int J Environ Res Public Health. 2020 Jul 1;17(13):4744. doi: 10.3390/ijerph17134744 (PMC7369814; doi:10.3390/ijerph17134744)
Supplement: Supplementary file 1 [file ijerph-17-04744-s001.zip › S3_Family Questionnaiere_Spanish.pdf]

OcioGune | resortes proyecto I+D+i
Red de Investigación en Ocio

MINISTERIO DE ECONOMÍA Y COMPETITIVIDAD

Unión Europea
Fondo Europeo de Desarrollo Regional

FINANCIADO POR EL PLAN NACIONAL I+D+i (2012-2015)

Códigos: **EDU2012-39080-C07-00**

EDU2012-39080-C07-01 | EDU2012-39080-C07-05
EDU2012-39080-C07-03 | EDU2012-39080-C07-06
EDU2012-39080-C07-04 | EDU2012-39080-C07-07

CUESTIONARIO PARA LAS FAMILIAS

De los tiempos educativos a los tiempos sociales: la construcción cotidiana de la condición juvenil en una sociedad de redes. Problemáticas y alternativas pedagógico-sociales.

Con este cuestionario, anónimo y confidencial, pretendemos conocer cómo influyen los tiempos académicos y de ocio en la vida cotidiana de los chicos y chicas que están cursando Educación Secundaria Postobligatoria. Solicitamos su colaboración para que nos dedique unos minutos y responda con sinceridad a todas las preguntas.

Por favor, una vez respondido, métalo en el sobre, ciérrelo y devuélvalo a su hijo o hija para que lo entregue en su centro de estudio; allí los cuestionarios serán recogidos por una persona del equipo de investigación.

Muchas gracias.

Persona que responde al cuestionario: ☐ Madre/ tutora ☐ Padre/ tutor

Año de nacimiento de la persona que responde al cuestionario: ....

SU HIJO/A (que le entregó el cuestionario)

1. **Desde que su hijo/a está en Educación Secundaria Postobligatoria** *(desde que dejó la ESO)*, **¿cuál es la nota que se repite más en sus evaluaciones?** Indique una cifra entre 0 y 10: ....

2. **Su hijo/a, además de estudiar, ¿tiene un trabajo remunerado?** ☐ Si ☐ No

DATOS GENERALES DE LA FAMILIA

3. **Indique el número de hijas o hijos que tiene a su cuidado en función de los siguientes tramos de edad** (sin incluir el hijo/a que le entregó el cuestionario):

Menos de 3 años	De 3 a 6 años	De 7 a 12 años	De 13 a18 años

4. **Aproximadamente ¿cuáles son los ingresos mensuales de su unidad familiar?** .... €

5. **¿Cuál es su nivel de estudios y situación profesional?** *Marque con una "X"*

Estudios				Situación Profesional					
Ninguno	Primarios (EGB)	Secundarios (Bachiller, FP)	Superiores Universitarios	Empleado/a cuenta ajena	Trabajador/a cuenta propia (autónomo, empresario)	Atención del hogar	Paro	Pensionista	Jubilado/a
☐	☐	☐	☐	☐	☐	☐	☐	☐	☐

TIEMPOS COTIDIANOS

6. **Indique su grado de acuerdo con las siguientes expresiones, escribiendo en cada casilla un número entre 1 y 5** *(1= nada, 2=poco, 3=algo, 4=bastante y 5=mucho)*

		1-5
El horario escolar condiciona la organización del tiempo de mi familia		
Nuestra familia está satisfecha con el horario escolar		
Los centros educativos deberían abrir sus instalaciones cuando no hay clases		
Mi hijo/a tiene una jornada diaria agotadora		
Estoy satisfecho/a con las actividades de ocio de mi hijo/a		
Las horas de sueño de mi hijo/a son suficientes		
El tiempo compartido en familia es…	suficiente	
	de calidad	
Donde vivimos…	hay oferta suficiente de actividades de ocio para los jóvenes	
	hay suficientes equipamientos de ocio para los jóvenes (casa de cultura, centro cívico, pabellón polideportivo, etc.)	
	hay suficientes espacios abiertos de encuentro (zonas verdes, parques, plazas, etc.)	
Sobre las vacaciones escolares…	su duración es suficiente	
	están bien distribuidas a lo largo del curso	

TIEMPOS COTIDIANOS

7. ¿Qué modalidad de horario escolar le parece mejor para su familia? *Tomando como referencia el horario del hijo/a que le entregó este cuestionario*

- ☐ Tener clase sólo por las mañanas
- ☐ Tener clase sólo por las tardes
- ☐ Repartir las clases entre todas las mañanas y una tarde
- ☐ Repartir las clases entre todas las mañanas y varias tardes
- ☐ Repartir las clases entre todas las mañanas y todas las tardes
- ☐ Te resulta indiferente
- ☐ Otra. ¿Cuál? ....

8. ¿Cuántos minutos dedica su hijo/a diariamente a estudiar...? .... minutos

Este tiempo… ☐ Es escaso ☐ Está bien ☐ Es excesivo

9. Indique el grado de preocupación que le producen las siguientes realidades en relación al hijo o hija que le ha entregado el cuestionario *(1= nada, 2=poco, 3=algo, 4=bastante y 5=mucho)*

	1-5		1-5
Las drogas		Falta de valores	
El alcohol		Competitividad	
Excesiva libertad		Falta de expectativas vitales	
Pocas obligaciones		Futuro laboral	
Consumismo		Fracaso escolar	
Falta de responsabilidad		Inseguridad y violencia en la calle	
Demasiado tiempo ante el televisor y otros dispositivos		Malas compañías	
Poco tiempo libre compartido en familia		Conductas delictivas (pequeños hurtos, tráfico de drogas, etc.)	
Soledad y aislamiento		Poco tiempo libre	
Falta de motivación			

TIEMPOS DE OCIO

10. Valore del 1 al 5 la importancia que otorga al ocio de sus hijos/as *(1= nada, 2=poco, 3=algo, 4=bastante y 5=mucho)*

Valoración: ....

11. Indique su grado de acuerdo con las siguientes expresiones *(1= nada, 2=poco, 3=algo, 4=bastante y 5=mucho)*

**Las actividades de ocio que realiza mi hijo hacen que…**	1-5
Esté más en forma, controle mejor sus movimientos, mantenga o mejore su condición física, etc.	
Se sienta más satisfecho, disfrute haciéndolas, se divierta, etc.	
Sea más creativo, aprenda a tomar decisiones autónomas, adquiera conocimientos, aprenda cosas sobre sí mismo y sobre los demás, amplíe su mundo, etc.	
Desarrolle nuevas destrezas manuales y adquiera o perfeccione habilidades técnicas	
Aumente su autoestima, se sienta más competente y capaz	
Haga cosas diferentes con más gente, se sienta parte de un grupo, se relacione mejor con los demás, etc.	

12. Indique con qué frecuencia realizan en familia las siguientes actividades (incluyendo al hijo/a que le entregó el cuestionario): *(marque con una "X" cuantas veces necesite)*

	Todas las semanas					Sólo 1-2 días al mes	Sólo en vacaciones	Nunca
	Lunes a viernes			Fin de semana				
	1-2 días	3-4 días	5 días	1 día	2 días			
Ver la televisión, vídeos, dvd, etc.								
Juegos de mesa (parchís, ajedrez, etc.)								
Jugar en el ordenador o con consolas								
Hobbies como fotografía, pintura, escritura, bricolaje, tocar instrumentos musicales, etc.								
Practicar actividades físicas y deportivas (andar en bici, caminar, nadar, etc.)								
Participar en asociaciones (de vecinos, culturales, deportivas, etc.)								
Asistir a espectáculos culturales (cine, teatro, conciertos, etc.)								
Asistir a espectáculos deportivos (partidos, torneos, etc.)								
Salir a comer o a cenar								
Viajar								
Otro ¿cuál?								

VIDA FAMILIAR

13. Indique su grado de acuerdo con las siguientes expresiones *(1= nada, 2=poco, 3=algo, 4=bastante y 5=mucho)*

	1-5
La mayoría de las veces mi vida familiar es ideal	
Las condiciones de mi vida familiar son excelentes	
Estoy satisfecho/a con mi vida familiar	
Hasta ahora he conseguido las cosas importantes que quiero en mi vida familiar	
Si pudiera decidir sobre mi vida familiar, no cambiaría nada	

SALUD Y CALIDAD DE VIDA

14. Indique cuántos días a la semana (de lunes a domingo) camina o pasea y hace deporte y cuánto tiempo le dedica

Paseo o camino .... días a la semana y suelo dedicarle .... minutos cada vez.
Practico deporte .... días a la semana y suelo dedicarle .... minutos cada vez

15. Indique su grado de acuerdo con las siguientes expresiones *(1= nada, 2=poco, 3=algo, 4=bastante y 5=mucho)*

	1-5
Estoy satisfecho/a con mi imagen corporal	
Estoy en buena forma física	
Soy una persona físicamente activa	
En general, estoy a gusto con mi vida	

ESTUDIOS Y MERCADO LABORAL EN EL FUTURO

16. Indique su grado de acuerdo con las siguientes expresiones *(1= nada, 2=poco, 3=algo, 4=bastante y 5=mucho)*

**Estudiar ayuda a mi hija/o a…**	**1-5**	**Trabajar ayuda a mi hija/o a…**	**1-5**
Tener éxito en la vida		Independizarse	
Encontrar trabajo		Colaborar con la familia	
Relacionarse con los compañeros/as		Sentirse útil	
Ganar dinero		Disponer de dinero	
Prefiero que mi hija/o busque trabajo a que siga estudiando		Merece la pena que mi hija/o busque trabajo	

EMPRENDIMIENTO

El **emprendimiento** se entiende como crear su propia empresa o autoemplearse, es decir, detectar una oportunidad de negocio, gestionar los recursos necesarios y asumir riesgos para contribuir a su desarrollo profesional y socioeconómico.

17. Valore del 1 al 5 en qué grado le gustaría que su hijo/a se decidiera a crear su propia empresa *(1= nada, 2=poco, 3=algo, 4=bastante y 5=mucho)*
Valoración: ....

18. Valore del 1 al 5 en qué grado ayudaría a su hijo/a si se decidiera a crear su propia empresa *(1= nada, 2=poco, 3=algo, 4=bastante y 5=mucho)*
Valoración: ....

**Mi ayuda sería...**	**1-5**
Apoyo económico	
Apoyo moral	
Aportando ideas	
Otro tipo de apoyo ¿cuál?	

Observaciones. Si quiere realizar alguna anotación utiliza el espacio siguiente:

MUCHAS GRACIAS POR SU COLABORACIÓN

Red de Grupos de Investigación OcioGune
Universidades
Santiago de Compostela, Barcelona, Burgos, Deusto, La Rioja, Nacional de Educación a Distancia, Valéncia.

Contacto: angela.devalenzuela@usc.es
